# The assessment of antiangiogenic and antivascular therapies in early-stage clinical trials using magnetic resonance imaging: issues and recommendations

**DOI:** 10.1038/sj.bjc.6602550

**Published:** 2005-05-03

**Authors:** M O Leach, K M Brindle, J L Evelhoch, J R Griffiths, M R Horsman, A Jackson, G C Jayson, I R Judson, M V Knopp, R J Maxwell, D McIntyre, A R Padhani, P Price, R Rathbone, G J Rustin, P S Tofts, G M Tozer, W Vennart, J C Waterton, S R Williams, P Workman

**Affiliations:** 1 On behalf of the Pharmacodynamic/Pharmacokinetic Technologies Advisory Committee (PTAC), Drug Development Office, Cancer Research UK, PO Box 123, Lincoln's Inn Fields, London WC2A 3PX, UK

**Keywords:** biomarker, magnetic resonance, therapeutic trials, antiangiogenic, antivascular, cancer therapy

## Abstract

Vascular and angiogenic processes provide an important target for novel cancer therapeutics. Dynamic contrast-enhanced magnetic resonance imaging is being used increasingly to noninvasively monitor the action of these therapeutics in early-stage clinical trials. This publication reports the outcome of a workshop that considered the methodology and design of magnetic resonance studies, recommending how this new tool might best be used.

Recent developments in our understanding of the cancer genome, and the control of molecular processes important to the development and regulation of cancer cells, are leading to the identification of many new targets for cancer therapeutics (Kohn *et al*, 2004). This increased understanding has been accompanied by new technologies that speed up the search for, and development of, new therapeutics (Workman and Kaye, 2002). Given the greater specificity of new agents against target processes, together with an expected growth in the number of such agents being proposed for clinical trial, the New Agents Committee (NAC) of Cancer Research UK, which approves and supports clinical trials of promising cancer therapeutics, is placing considerable emphasis on incorporating end points that provide evidence that the desired activity has been achieved into new trials (Newell *et al*, 2003). To assist in this, the Pharmacodynamic/Pharmacokinetic Technologies Advisory Committee (PTAC) reviews submissions to the NAC for new therapeutics being considered for further development or clinical trial, to identify and advise on the incorporation of pharmacodynamic and pharmacokinetic assessment into early-stage clinical trials (http://science.cancerresearchu k.org/tcr/drugdevelopment/newa gentscomm/ptac). Where possible, PTAC is looking for trial designs that test whether the therapy is working by its intended process (Workman, 2002, 2003). Guidelines and advice are available at http://science.cancerresearchu k.org/reps/pdfs/PTACguidelines .pdf. While morphological tumour size change remains an important measure of response, in early-stage trials, or in the evaluation of cytostatic or antiproliferative agents, volume change may not be a sensitive-enough measure of the biological activity of an agent. In the setting of early-stage trials performed on patients with advanced and refractory disease, treatments may affect a proportion of cells but not translate into volume reduction. Therapeutics targeted at tumour vasculature or its control provide an example of a class of treatments where functional imaging may provide earlier or more specific information than morphological imaging.

Tumour vasculature presents an important target for therapeutic development, with agents acting on the vascular endothelium (antivascular therapies) (Thorpe, 2004) or the process of neoangiogenesis (antiangiogenic therapies) (Bicknell and Harris, 1996). Magnetic resonance imaging (MRI) has shown promise as a method of evaluating tumour vasculature, potentially monitoring the effect of these therapies (Taylor *et al*, 1999; George *et al*, 2001; Padhani and Husband, 2001; Padhani *et al*, 2001; Galbraith *et al*, 2002, 2003; Morgan *et al*, 2003; Neeman and Dafni, 2003). The PTAC has recently considered a number of applications to evaluate these classes of therapeutic agent, with proposals to employ MRI to assess activity. In considering these proposals, it was clear that investigators would be helped by guidelines defining requirements for MRI investigations in early-stage trials of antivascular and antiangiogenic therapies. To this end, PTAC convened a workshop to address these issues, and to provide appropriate guidelines. The workshop considered the types of therapeutic and their effects; the requirements of clinicians and industry; the potential MRI approaches, what they measured and the reproducibility of the techniques; the approaches taken in alternative methods of vascular evaluation. Several different panels, detailed in the Appendix, comprised of the authors of this report, then considered the various specific and important issues involved in applying MRI to antivascular and antiangiogenic therapies, and developed guidelines for such use. This report summarises the presentations and conclusions of the panels, together with the recommended guidelines.

## THERAPEUTIC DEVELOPMENT, ANTIVASCULAR AND ANTIANGIOGENIC THERAPIES (BOXES 1 AND 2)

In building on the increasing pace of target discovery and the development of potential therapeutics, there is a need to evaluate the effects of drugs on their biological targets *in vivo*, and to perform clinical trials more effectively. Some of the important questions are listed in Box 1.

Pharmacokinetic assays such as liquid chromatography–mass spectrometry (LC–MS) can define plasma concentrations of agents. However, some of the powerful pharmacodynamic assays that assess biological effect, such as array technology or immunohistochemical methods, require tissue and are surgically invasive. This raises ethical and logistical difficulties in incorporating these assays into clinical trials. This is particularly the case where the timing of peak biological effect post-treatment is not known. Noninvasive imaging methods are more acceptable, are generally not limited to biopsy-accessible sites and may permit more frequent measurements.

There is a wide range of compounds that affect vascular function (Li, 2000). Their effects are summarised in Box 2. Antiangiogenic agents (Verheul and Pinedo, 2003; Alessi *et al*, 2004; Cao, 2004) include inhibitors of vascular endothelial growth factor (VEGF), receptor tyrosine kinase inhibitors, inhibitors of other receptors and signalling pathways (e.g. FLT1, PI3 kinase, HIF) and angiostatin-like agents. These agents are directed at inhibiting or disrupting the growth of tumour neovasculature. In addition, a wide range of other agents, including inhibitors of matrix metalloproteinases, anti-cytokines and general cytotoxics, may have vascular-directed effects. Examples of antiangiogenic agents include vatalanib, bevacizumab, ZD6474, SU11248, AGM-1470 and PTK787/ZK222584. Antivascular agents (Siemann *et al*, 2004; Thorpe, 2004) include Combretastatin A4 phosphate, ZD6126 and 5,6-dimethylxanthenone-4-acetic acid (DMXAA). These are directed against the vascular endothelium, resulting in collapse of tumour vasculature.

## WHAT CAN MRI MEASURE?

Magnetic resonance imaging methods provide an attractive means of investigating vascular end points since they are widely available, noninvasive and involve no ionising radiation. At the simplest level, MRI gives anatomical information, with the powerful potential to manipulate the relative contrast of different tissues. In practice, the MRI method that is almost invariably used for vascular studies is dynamic contrast-enhanced MRI (DCE-MRI). This involves injection of a contrast agent and acquisition of a series of images; the curve of signal intensity change indicates the rate of uptake of the contrast agent into the tumour and its subsequent washout. Dynamic contrast-enhanced MRI is widely used in radiological practice for diagnostic purposes, and in this role interpretation usually involves subjective judgements about the shape of the uptake curve and the morphology of enhancing tissues. In drug trials, quantifiable parameters aid assessment of change and of dose-related effects. A range of techniques to make quantitative measurements have been reported (Collins and Padhani, 2004), allowing parameters descriptive of vascular physiology and processes to be calculated. However, the physiological significance of these parameters can be complex, and there is currently no consensus as to the best parameters to be used, or the most appropriate measurement and analysis methodology.

Magnetic resonance imaging predominantly images water molecules in the body. By exploiting specific properties of magnetic resonance and of water, a number of approaches to assessing tissue vasculature and vascular properties have been developed. These provide methods of assessing antiangiogenic and antivascular therapies. In an MRI investigation, these functional measurements can be combined and registered with high-quality morphological images, allowing conventional size assessment, identification of heterogeneity and visualisation of adjacent structures.

Magnetic resonance methods of assessing vasculature and vascular properties can be divided into:

1. visualisation and assessment of vascular structures including MR angiography
2. assessment of vascular delivery of molecules to the extravascular space
3. measurement of blood volume and
4. measurement of perfusion.

Magnetic resonance contrast agents utilise atoms or compounds with magnetic properties that will affect hydrogen atoms in water molecules, changing the signals measured. The most common agents utilise chelates containing paramagnetic atoms such as gadolinium (Gd), which have one or more unpaired electrons, resulting in a strong electronic magnetic moment. This local magnetic field can alter (relax) the magnetic state of the hydrogen atoms in water, changing their T1 relaxation time (an MR parameter describing their response to MR measurements) causing an increase in signal on T1-weighted MR images. Strong concentrations of Gd-containing contrast agents can also change the local magnetic field (changing magnetic susceptibility). This causes a local loss of signal due to dephasing, which is described by T2^*^ relaxation, and can be seen as a loss of signal on T2^*^-weighted sequences. The paramagnetic atom is chelated in a compound that will define the pharmacokinetic behaviour of the compound in the body. Most agents currently in clinical use are low-molecular-weight compounds (MW<1000), without specific targeting properties, but which can diffuse through normal endothelial junctions (Schneider and Uder, 2003). However, more complex high-molecular-weight agents are being developed, some of which may act as blood pool agents, and others may be substrates for specific processes (Brasch and Turetschek, 2000; Turetschek *et al*, 2004). A particularly important feature of all these approaches is that the contrast agent is not being measured directly, rather it is the effect that it has on the very many water atoms that can visit the Gd that is measured, effectively amplifying the effect of each molecule of contrast agent.

**Figure 1:**
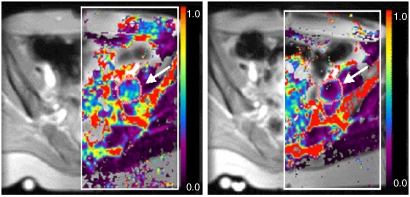
Example of *K*^trans^ maps, superimposed on T1-weighted images of a patient with primary peritoneal carcinoma with left pelvic side wall nodal metastases (arrow). Images are before and 4 h after the first dose of Combretastatin (52 mg m^−2^). The colour scale ranges from *K*^trans^ values of 0 to 1 min^−1^. A dramatic reduction of transfer constant is seen. This was published with kind permission from the Journal of Magnetic Resonance Imaging (Padhani, 2002).

Conventional MR imaging compares similar images with and without the contrast agent. Thus compared with precontrast images, T1-weighted images after contrast will show bright areas where the Gd-containing contrast agent has been delivered to the extracellular space. This approach is routinely used in MRI, particularly in the brain where uptake of Gd shows breakdown of the blood–brain barrier. Usually, such images are taken at a few minutes after contrast administration, but in some tissues, it is important to make measurements at shorter time periods, as the contrast agent can washout rapidly. More recently, there has been interest in following the dynamic uptake of the contrast, and a set of rapid images have been used, allowing the time course of intensity changes to be measured in a region of interest (ROI). This can provide diagnostic information, for example in the diagnosis of breast cancer (Leach, 2001). To cause enhancement, the agent must be delivered to the tumour, and must leak into the extracellular space, providing information on perfusion, on endothelial permeability and on the volume of the extracellular, extravascular space. This approach is particularly sensitive to developing neovasculature in tumours, which is dependent on VEGF and other growth factors generated by primary and metastatic tumours (Choyke *et al*, 2002; Strecker *et al*, 2003). Analysis of the dynamic image series can be used to calculate parametric maps of pharmacokinetic parameters, or of parameters based on physiological models, with a spatial resolution of typically 1–2 mm (see Figure 1). Definitions and terms used in parametric measurements are defined in Box 6.

When a bolus of contrast agent passes through the vasculature, having a high concentration of contrast, it changes the magnetic susceptibility of the blood, causing large magnetic field gradients compared with surrounding tissues. This results in local signal loss on T2^*^-weighted images, which can provide information on vascular concentrations of agent, but can also generate information on vessel dimensions, as the size and extent of the effect is dependent on vessel diameter. Sequences that have a different sensitivity to vascular dimensions (such as spin-echo or gradient-echo sequences) can also be used to probe vascular size. In addition, blood provides an intrinsic contrast, with the degree of oxygenation affecting signal intensity on T2-weighted sequences. Oxyhaemoglobin is diamagnetic, whereas deoxyhaemoglobin is paramagnetic. Thus, deoxygenated blood causes more signal loss on T2^*^-weighted images, a phenomenon exploited in blood oxygen level-dependent (BOLD) imaging (Howe *et al*, 2001) and brain activation studies (Hennig *et al*, 2003). This approach can be further exploited by using vasomodulators such as carbogen, which will preferentially affect physiologically responsive mature vasculature. These techniques probe vascular structure at a resolution below that of the imaging method. Many trials of new antiangiogenic or antivascular agents already include DCE-MRI measurements to provide biomarkers of therapeutic action (Galbraith *et al*, 2002, 2003; Jayson *et al*, 2002; Morgan *et al*, 2003; Yang *et al*, 2003). In principle, some of the other methods described above can be included in such studies to increase the information obtained.

## CLINICAL VIEW (BOX 3)

Increasingly, early-stage clinical trials are being designed to provide information on biomarkers of therapeutic effect, in addition to establishing pharmacokinetics, safety and tolerability for single agents and combinations. Evidence from these trials that agents are acting on their intended target increases confidence in the value of taking them through to larger stage trials of efficacy, or can inform further development. Some issues relating to clinical application of MR techniques are summarised in Box 3. Techniques used in hypothesis testing trials need to be robust, practicable in a clinical setting and acceptable in terms of invasiveness. Approaches need to be properly evaluated and be compatible with ICH Good Clinical Practice (ICH, 1996).

Antiangiogenic agents are expected to be most effective on immature new vasculature, with small emerging tumours most likely to exhibit neoangiogenesis and so respond to such treatment. Thus, the maximum benefit might be seen in an adjuvant setting. Imaging techniques are, however, best suited for evaluating larger tumours that can be clearly resolved and separated from other tissue structures. Patients chosen for early-stage clinical trials often have received multiple treatments or have stable metastatic disease, resulting in a mature vascular phenotype. Early-stage clinical trials on advanced disease therefore may not provide evidence of volume response, but changes in vascular function in defined areas of tumour may support the hypothesis under test. Robust statistical techniques may be needed to identify these effects. Sufficiently sensitive techniques may indicate whether the agent has an impact on a proportion of the tumour vasculature. Some metastases may provide imaging targets with a high proportion of neovasculature. It will be important to separate direct vascular effects of treatment from vascular change resulting from cytotoxic action on tumour cells. Later stage trials may, however, require evidence of an improvement in progression-free survival rather than of an arrest of progression.

Antivascular agents, such as cytoskeleton-interacting drugs, cause acute vascular collapse. These effects seem to be rapidly reversible, but may have less effect on more mature vessels. They may be most effective as combination therapy with cytotoxics or antiangiogenics. To move these agents through to clinical use, it is necessary to establish that they cause clinically useful disease stabilisation. As bulk tumour response may be hard to obtain in advanced disease, surrogates such as biopsy studies or imaging may be required to define an effective dose and provide sufficient confidence to progress to phase III trials.

## PHARMACEUTICAL INDUSTRY VIEW (BOX 4)

The requirement for identifying imaging end points for therapeutic evaluation comes from the imperatives of portfolio management. There are many potential targets to choose from, and multiple possible molecules to affect each target. These cannot all be taken through to stage III therapeutic trial. Thus, it is necessary for the pharmaceutical industry to rationalise the development process to minimise the costs of developing new agents, while at the same time accelerating drug development and minimising patient exposure to ineffective drugs/doses. In this context, the main interest in functional imaging end points are in phase I/IIa trials with the objectives of obtaining an acute marker of biological efficacy (ideally pharmacodynamic), identifying the biologically effective dose, which may be much less than the maximum tolerated dose (MTD), and evaluating scheduling options and possible combination therapies. In this context, it is important to note that MR-measurable effects, which report on aspects of overall vascular function (see section What can MRI Measure), may not strictly reflect the concentration of the drug (due to the complexity of vascular function and control, and the time course of treatment effects). Major issues are summarised in Box 4.

Industry places considerable emphasis on studies delivering appropriate data. The imaging study must be feasible. Repeat scans should be practicable and acceptable to patients. The method should be implementable in a multicentre study. It should be feasible in all the chosen and relevant anatomical locations especially in the advanced disease typically encountered in phase I/II trials. Analysis should be robust and reliable. The approach should be thoroughly evaluated and be widely accepted. There should be a well-understood link to the underlying molecular pharmacology in order to distinguish alternative mechanisms. It should be likely to predict clinical response, but may not necessarily be accepted by regulatory bodies such as the US Food and Drug Administration (FDA) as a valid surrogate end point. The measurement method should be sufficiently well understood for a clear primary end point to be established prospectively. It should provide good and known statistical power throughout the dose–response curve, with potential confounds being understood and controlled. While it was thought desirable that the measurement and analysis process should meet the requirements of ICH Good Clinical Practice (GCP) (ICH, 1996), there is recognition that the investment of resources required for the validation of computer software, which is extremely onerous, might be unrealistic, and could inhibit the imaginative use of novel techniques. The other extreme would be to work to ‘publication standard’, using investigator written software. However, this requires maintenance of expertise in the software, implying long-term investment by the investigator's institution in this capability. Ideally, some robust medium position between ICH GCP and ‘publication standard’ is required for the analysis, together with mechanisms for funding, maintaining and sharing the necessary measurement, evaluation and quality assurance methods and resources.

Prior to a clinical trial, the practicalities need to be addressed. The methodology should be developed, established and validated beforehand, and animal studies to inform study design should have been performed. The method of identifying and delineating the lesion needs to be defined, including who will draw the ROI or volume of interest (VOI), and how many lesions will be measured. The type of analysis must also be specified, clearly indicating the methods used. Standard radiologist's imaging reports can readily identify powerful drug effects but cannot provide a basis for establishing a dose–response relationship; nonpharmacokinetic quantification such as numerical descriptors of uptake-curve shape (e.g. maximum intensity time ratio (MITR); some examples are given in Brown *et al*, 2000) is based on signal intensity changes and may not translate between centres. Parameters may be determined from standard pharmacokinetic models (*K*^trans^…); from more advanced pharmacokinetic models, including vascular input functions; or from integration of the contrast agent concentration curve (IAUGC). Standard terms are defined in Box 5. The quantification needs to be fit for purpose, and should include the generation of information that is necessary to robust characterisation and intercentre consistency, such as the input function or the native T1 relaxation time. Issues relating to heterogeneity need to be addressed, such as whether single-slice or three-dimensional (3D) analyses are required, whether model fitting should be performed on ROI data or pixelwise. If pixel-by-pixel analysis is performed, should such analysis use the median, or should more complex analysis based on the histogram of values within the lesion be employed? This should be established and documented prospectively.

The limitations of animal models in defining the size of effects to be expected, as well as their timing, were considered. Such measurements were viewed as important in functional imaging studies, both to give guidance on the timing and maximum effect, and also to aid in the interpretation of clinical results. Data should be capable of independent review, but should not be expected to reach a standard that could not be attained at most centres, as this could adversely affect the introduction of these new approaches. There is concern as to who would pay for the validation of these new methods, as there is a tension between the requirements of academic research funders who fund new science and the requirements of individual pharmaceutical companies who wish to support development of their own agents, desiring computer system validation in compliance with ICH GCP standards to support decisions that have major financial implications.

## RECOMMENDATIONS FOR MR MEASUREMENT AND ANALYSIS METHODS, END POINTS AND NOMENCLATURE FOR USE IN PHASE I/IIA TRIALS OF ANTICANCER THERAPEUTICS (BOXES 5 AND 6)

### Measurement methods

When MRI is used for the pharmacodynamic assessment of antivascular and antiangiogenic agents, it is recommended that DCE-MRI should be performed with low-molecular-weight Gd chelates, because of the well-established sensitivity of this approach to perfusion and endothelial permeability. Noncontrast MRI measurements may be included in addition to DCE-MRI. Magnetic resonance imaging measurements with high-molecular-weight contrast agents are expected to add a great deal to the quantitation and specificity of these measurements, but high-molecular-weight agents are not yet generally available for use in phase I/IIa trials of anticancer therapeutics. Dynamic contrast-enhanced MRI is widely used in radiology. If a compartmental model is assumed, kinetic parameters from DCE-MRI studies can be assessed by quantifying contrast agent concentration change or ΔR1 (i.e. the change in 1/T1) and then using pharmacokinetic modelling techniques. An alternative quantitative approach, which does not assume any particular model, is the initial area under the Gd concentration–time curve, IAUGC. Some investigators however have relied on the semiquantitative analysis of signal intensity changes. Semiquantitative parameters have the advantage of being relatively straightforward to calculate, but have significant limitations. These limitations include the fact that semiquantitative parameters may not accurately reflect contrast agent concentration in the tissue of interest and can be influenced by the contrast agent injection procedure and the scanner settings (including the pulse sequence, gain and scaling factors) and target position in the image. These factors limit the usefulness of semiquantitative parameters because between-patient and between-system comparisons are difficult. It is also unclear what these parameters reflect physiologically and how robust they are to variations of cardiac output. As such, semiquantitative parameters are not recommended for the evaluation of antiangiogenic/antivascular clinical trials. In general, quantitative measurement techniques are used when modelling tissue contrast agent concentration although some workers model signal intensity, making the assumption that signal intensity changes are proportion to contrast agent concentration changes (Brix *et al*, 1991; Buckley *et al*, 1994; Hoffmann *et al*, 1995). Quantitative T1 changes measured during a dynamic enhancement acquisition can be used to estimate contrast agent concentration *in vivo* (Brookes *et al*, 1996; Parker *et al*, 1997, 2000). Concentration–time curves are then mathematically fitted using one of a number of recognised pharmacokinetic models (Tofts *et al*, 1999) and quantitative modelling parameters are derived. Standardised terms should be used as defined by Tofts *et al* (1999) (see Box 5).

Magnetic resonance measurements to address the primary end points need methods capable of rapidly measuring contrast agent concentration, by assessing tumour T1 relaxation times. This requires an estimate of the effective relaxivity of the chosen contrast agent in tumour vasculature and tissue. This relaxivity may be different from the relaxivity in pure water, because of water exchange and protein binding. To calculate contrast agent concentration, measurements also need to assess the native T1 of the tissue. This requires the use of quantitative imaging techniques, which need to be validated against test objects. Many methods are available for the assessment of T1, but the faster methods (progressive nutation) are vulnerable to error due to radio frequency field inhomogeneity and mis-set pulses (Gowland and Stevenson, 2003; Parker and Padhani, 2003). The accuracy of T1 measurements should be verified for every spatial location, coil and MR scanner deployed in the study. This implies incorporation of an appropriate quality assurance programme, which is particularly important for multicentre trials.

Recommendations for the associated nomenclature and analysis approaches are provided in Box 5. Recommendations for measurement methods and end points are summarised in Box 6.

### Primary end points

It is recommended that the primary end points in phase 1/2a MRI trials of oncology therapeutics should be either *K*^trans^ (min^−1^) or IAUGC (mM Gd min).

*K*^trans^ or transfer constant is the most widely accepted kinetic parameter, describing the transendothelial transport of low-molecular-weight contrast medium into the extravascular–extracellular space by diffusion (Tofts *et al*, 1999). The interpretation of *K*^trans^ is dependent on the tissue being evaluated and on the underlying physiological circumstances. If the delivery of the contrast agent to a tissue is insufficient (flow-limited situations or where vascular permeability is greater than inflow), then blood perfusion will be the dominant factor determining contrast agent kinetics and *K*^trans^ approximates to tissue perfusion per unit volume (Taylor *et al*, 1999; Tofts *et al*, 1999). If tissue perfusion is sufficient and transport out of the vasculature does not deplete the intravascular contrast agent concentration (non-flow-limited situations, i.e. permeability-limited), then transport across the vessel wall is the major factor that determines contrast agent kinetics. In tumours, the truth is likely be somewhere between the two. We can say that the transfer constant is approximately equal to the smaller of the permeability surface area product (PS) or perfusion (*F*), that is, *K*^trans^≈min(PS,*F*). *K*^trans^ is obtained from simple compartmental models (Tofts *et al*, 1999). Although *K*^trans^ is a complex function of tumour blood flow, endothelial surface and endothelial permeability (Tofts *et al*, 1999), an effective agent will be expected to reduce some or all of these fundamental physiological parameters and it should therefore decrease *K*^trans^.

IAUGC is a phenomenological description of the early part of the Gd uptake curve (Evelhoch, 1999). IAUGC does not require any curve-fitting, or knowledge of an accurate physiological model, and so is expected to be more robust than *K*^trans^, which can be vulnerable to fit failures in the case of highly vascular regions, very poorly perfused regions or physiological motion. It is calculated from the area under the contrast agent concentration curve up to a specified cutoff time (usually 60 s). This parameter must be derived from the contrast agent concentration (not signal intensity) curve. Thus the time-varying T1 measurement change must be measured. IAUGC may be more reliable in the presence of noise or large intravascular tracer concentration than other quantitative parameters, but as for pharmacokinetic modelling parameters, it requires measurement of quantitative T1 changes. The relationship between IAUGC and underlying physiology is complex and undefined (Larcombe-McDouall *et al*, 1991; He and Evelhoch, 1998). Both IAUGC and *K*^trans^ can be used to derive a ‘vascularised tumour volume’ by counting the voxels with values above a predetermined threshold.

It is recommended that the vascular Gd concentration–time curve (ideally, the tumour arterial input function) should be determined for each patient. The method and site of vascular measurement should be appropriate to the tumour under investigation. This is an area of current research, and may involve direct arterial measurements, or generation of a normalisation factor. Where this is not possible, steps should be taken to control for differences in the input function either due to differences in bolus injection, where use of a power injector is advised, or due to changes in cardiovascular or renal function. The patient's cardiovascular function should be monitored and recorded, and consideration should be given to measuring stroke volume, heart rate and/or Gd uptake in normal tissue. These parameters can be used to provide some normalisation for changes in arterial input to a tumour.

IAUGC or *K*^trans^ measurements should be made for each voxel in the ROI or VOI. Ideally, 3D assessment of the entire tumour should be made, since single-slice 2D assessments are (at least in theory) prone to bias. Tumour volume should be assessed.

### Secondary end points from DCE-MRI

Simplified methods of characterising DCE-MRI data, such as slope, or time to maximum, have been reported and may be employed as secondary end points. However, there is insufficient information on the statistical power of these simplified approaches, and *a priori* the reliance on a small portion of the DCE-MRI curve may make them less sensitive than the use of *K*^trans^ or IAUC. Also they may be difficult to compare between centres. Other secondary end points from compartmental models may also be employed. The use of more elaborate models to derive secondary end points is encouraged, although regard should be paid to the possibility that complex models may not yield mathematically unique solutions.

*v*_b_: Methods are available to estimate *v*_b_ although more evaluation is required. Blood volume can be estimated from dynamic T2^*^-weighted imaging (Sorensen *et al*, 1997), although this usually precludes the simultaneous estimation of IAUGC or *K*^trans^. Alternatively, simultaneous T1 and T2^*^ data collection techniques can be used (Barbier *et al*, 1999; Zhu *et al*, 2000; d'Arcy *et al*, 2002). Recently, T1 measurements combined with modelling methods have been developed for estimating brain blood volume and permeability based on first-pass kinetics (Hacklander *et al*, 1996, 1997; Ludemann *et al*, 2001; Johnson *et al*, 2004). Roberts *et al* (2000) have described a slow time resolution technique for estimating blood volume and permeability in the brain. Such T1-weighted techniques have a variable correlation with conventional T2^*^-weighted techniques when estimating blood volume (Hacklander *et al*, 1997; Zhu *et al*, 2000).

*v*_e_, extracellular extravascular volume, may be employed as a secondary end point, although there is insufficient evidence that *v*_e_ provides an unbiased estimate of interstitial space, particularly with noisy data in poorly perfused regions. Also, effective agents might plausibly act either to increase or to decrease interstitial volume fraction, making this an unreliable parameter for decision-making.

*k*_ep_: Similar considerations apply to *k*_ep_ (previously *k*_21_), which reflects transfer of contrast from the extracellular space to the plasma. It can be measured using low time resolution measurements, and can also be derived from *K*^trans^/*v*_e_. Initial methods were based on an assumption that signal intensity was proportional to contrast agent concentration.

PS and *v*_p_: Endothelial permeability (PS) and the fractional plasma volume (*v*_p_) can be derived from the use of macromolecular contrast agent (MMCM)-enhanced DCE-MRI (without the flow contamination that can occur for *K*^trans^ measured with current low-molecular-weight ECF chelates (MW<1 kDa)). *v*_p_ can also be obtained from *v*_b_ with a knowledge of haematocrit. The advantages of using MMCMs include physiological relevance. Disadvantages include lack of evaluation in human subjects, current nonavailability for human use, signal-to-noise issues and the longer scanning time required to estimate PS by monitoring the loss of tracer from tumour.

### Secondary end points from non-contrast-enhanced MRI

Tumour T1, T2, T2^*^ relaxation times and the apparent diffusion coefficient (Wheeler-Kingshott *et al*, 2003) may provide useful measures of change. Perfusion using arterial spin tagging is of interest as a secondary end point, although it has limited signal to noise, and quantification is particularly demanding (Parkes and Detre, 2003). Magnetisation transfer is sensitive to water molecules bound to macromolecules and to cell membranes, and may provide additional information about tissue changes (Tofts *et al*, 2003b).

### Pharmacokinetic models

Three major models have been reported for leakage studies (Larsson *et al*, 1990; Brix *et al*, 1991; Tofts *et al*, 1995). They are reconciled by Tofts (1997). These models have been widely used, and allow well-understood comparisons between studies to be made. However, as with most models, they make a number of assumptions. Generally, an assumed plasma washout curve is used, based on the published Weinmann curve (Weinmann *et al*, 1984), reflecting the change in plasma concentration with time due to exchange and renal excretion. The Wienmann curve does not provide information on the short-term vascular bolus and the effects of bolus administration and vascular output on this. It assumes normal renal function. Use of a measured arterial input function can provide information on the short-term bolus delivered to the tissue, the effects of cardiac output and longer term exchange processes described by the plasma washout curve. Experimental methods to measure this and incorporate it into models are not yet common practice but are now being explored. This in principle should allow for changes in physiology and bolus administration, leading to increased reproducibility. The models do not allow for intravascular tracer, which can be a major component in tumours. The Brix model (Brix *et al*, 1991) assumes that vascular leakage is permeability limited and that signal is proportional to concentration. It also determines plasma decay from the fit. Information on blood volume *v*_b_ and perfusion can be obtained from T2^*^–weighted bolus tracking measurements. Models should calculate estimates of uncertainty by monitoring fitting and *χ*^2^; if the fit is poor for a given pixel or ROI, this should be labelled as a model failure and excluded from further analysis (e.g. ROI mean or histogram). The stability of fits with many parameters should also be considered.

### Analysis of regions of interest (ROI and VOI) (Box 7)

Most dynamic MRI studies utilise user-defined ROIs from image slices or VOIs from 3D data sets. The ROI and VOI methods yield graphical outputs with good signal-to-noise ratio, but lack spatial resolution and are prone to partial volume averaging errors. In its simplest form, an ROI or VOI encompassing the whole tumour cross-sectional area or volume is drawn, from which an average enhancement curve is extracted. Whole ROI analysis ignores heterogeneity of tumour enhancement by assuming that the averaged kinetic parameter estimate equates to those that would be obtained from individual pixels. Recently, Hayes *et al* (2002) have shown in primary breast cancer that whole ROI analysis can be a close approximation to individual pixel evaluation. However, many authors have commented that whole tumour VOI assessment may be inappropriate, particularly for the evaluation of malignant lesions where heterogeneous areas of enhancement are diagnostically important (Aronen *et al*, 1994; Gribbestad *et al*, 1994; Parker *et al*, 1997). Therefore, for some purposes, selective sampling of regions within a tumour is used based on the premise that the discrimination of lesions is improved by this approach (Liney *et al*, 1999). For evaluation of therapeutic response, selective sampling may not be appropriate, unless good *a priori* criteria for region selection can be developed.

Another approach for displaying dynamic data is by pixel mapping (Parker *et al*, 1997). This method depicts kinetic enhancement information as maps spatially coregistered with anatomical images on a pixel-by-pixel basis. This type of display has a number of advantages including the appreciation of heterogeneity of enhancement and avoidance of the need to selectively place user-defined ROIs. The risk of missing important diagnostic information and of creating ROIs or VOIs that contain more than one tissue type is reduced. An important advantage is the ability to spatially match tumour vascular characteristics such as blood volume, perfusion, transfer constant and leakage space. Regional differences in the distributions of kinetic parameters have been shown in xenografts (Bhujwalla *et al*, 2001) and human tumours (Roberts *et al*, 2000; Zhu *et al*, 2000; Ludemann *et al*, 2001). Such displays provide unique insights into tumour structure, function and response. As they avoid potential selection bias in determining local ROIs or VOIs, they may have advantages for monitoring treatment response to new agents. They may also aid guidance of biopsy sampling of tumours.

Pixel mapping techniques have the disadvantage of lower signal-to-noise ratios, and they also require specialist software for their generation (Hoffmann *et al*, 1995; Parker *et al*, 1998; Li *et al*, 2000). While visual appreciation of heterogeneity is improved by pixel mapping displays, quantification can be difficult. Recently, histogram analysis (Tofts *et al*, 2003a) has been used to quantify the heterogeneity of tumours for comparative and longitudinal studies, for monitoring the effects of treatment and to show the regression or development of angiogenic hot spots (Mayr *et al*, 2000; Hayes *et al*, 2002). Recommendations regarding the analysis of ROI or VOI are summarised in Box 7.

## STANDARDISATION, VALIDATION AND REPRODUCIBILITY (BOX 8)

To support the use of MR parameters in decision-making with regard to pharmaceuticals, it is important to show correlations between specific biological effects, their magnitude and the relevant MR parameter. This should be firstly performed in well-defined model systems, and then, where possible, confirmed by clinical measurements using tumour biopsy or if appropriate, surrogate tissues, taking into account the expected time course of activity. The biological end points ideally should relate specifically to the proposed mechanism of action of the compound. To allow appropriate study design, and to assess the significance of change, centres should demonstrate the reproducibility of their clinical measurements, in a manner that is traceable, providing information on individual and intergroup reproducibility. This information should be combined with evidence of the expected magnitude of therapeutic effect, such that the design can enable assessment of dose-related changes. Such assessment can be facilitated by incorporating baseline repeat measurements to provide information directly relevant to the body sites chosen.

It is recommended that standardised methods of analysis, as discussed in the previous section, be used. In order to allow comparison of different centres' analysis methods, and to enable assessment of new models, there is a need for standardised data sets to be made available. These should be documented, anonymised and be widely accessible (for example, via the world wide web). It would be advantageous if companies and academic investigators conducting trials could make data available in this form. Similarly, it would be desirable for research groups to make their analysis methods available either by publication of open code, or under specific sharing agreements. This policy would benefit the whole user community. In the longer term, specific standardised software for analysis would be advantageous, but this should not restrict the continual evolution of measurement and analysis approaches. Standard methods of T1 assessment should be established and validated against phantoms appropriate to specific body locations, with their measurement reproducibility being established. It is thought that there is limited value in direct comparison of different methods, which is only supported where there is likely to be a clear improvement. In these cases, comparison of IAUGC and/or *K*^trans^ is required as a minimum. Where multiple sites are participating in a trial, the methodology should be standardised and confirmed using imaging phantoms and a quality assurance programme. Data analysis should ideally be performed centrally, using validated software. Reliability of this analysis should be assured using data from each participating centre prior to starting the trial. Recommendations for standardisation, validation and reproducibility are summarised in Box 8.

## FUTURE DEVELOPMENTS (BOX 9)

MRI methods for use with antivascular and antiangiogenic drugs are still in their infancy, and rapid progress is likely in the next few years. There is an urgent need for further developments to facilitate the use of DCE-MRI. An underlying requirement is the need for a rapid and robust method for measuring T1, to allow contrast agent concentration to be estimated from measured signal intensity (Parker *et al*, 2001; Gowland and Stevenson, 2003). Rapid T1 measurement methods should be implemented by instrument manufacturers, together with standard protocols for calibrating and testing them. Correction for variation in the plasma concentration of contrast agent has been shown to be an important source of variation between measurements, limiting reproducibility. Measurement of arterial input function or generation of a normalising function based on nearby normal tissues are methods that are under development. The value of these approaches needs to be defined, and a standardised method developed and incorporated into analysis techniques. Improved statistical analysis techniques suitable for interrogating the parameter values within an ROI, which may define the whole tumour, are required. Current techniques employing simple descriptors of the histogram of parameter values, although more sensitive than simple ROI analysis, may not be optimised to detect the changes caused by a new agent in a phase I trial. Recommendations for future development are listed in Box 9.

In the longer term, there is a need for improved contrast agents, either with higher molecular weight or with functionally specific properties, to provide a read-out of direct therapeutic action. Measurements at many sites in the body are compromised by tissue motion, or by the physiological effects of nearby tissue motion. While sophisticated methods have been applied to, for example, cardiac imaging, these are generally not immediately applicable to other organs or tissues. Motion compensating or correcting techniques, which preserve the timing and quantitative nature of contrast measurements, are required, together with appropriate registration techniques. A range of other physiological and metabolic parameters can be measured that may inform the action of new therapeutics, and may aid discrimination of different tissue types within tumour. There is likely to be value in applying these additional approaches to tumour measurements, and developing appropriate combined analyses.

## Figures and Tables

**Box 1:** Issues relating to therapeutic development

Important to know if the drug is:
• Reaching required concentration in the effecting compartment.
• Affecting desired molecular target.
• Modulating the biochemical pathway.
• Achieving desired biological effect on the tumour cell.
• Achieving desired physiological effect on the tumour.

There is a requirement for noninvasive assays, particularly when timing of peak biological activity is not known.

Aim for hypothesis testing trials to:
• Support go/no go decisions: accelerate drug development or kill failures early.
• Establish dose and time response (scheduling).
• Examine drug combinations.
• Provide confidence to go forward to expensive large-scale trials.

In this context, MR measurements are biomarkers, where, following the NIH Biomarkers Definitions Working Group (2001), a biomarker is a ‘characteristic that is objectively measured and evaluated as an indicator of normal biological processes, pathogenic processes or pharmacologic responses to a therapeutic intervention’ as distinct from a surrogate end point, which, when validated, is expected definitively to predict clinical benefit. Surrogate end points generally require evidence of validation for regulatory bodies.
Aim to operate the analysis at a level of computer software validation between publication standard quality assurance and ICH GCP (1996).
Have realistic guidelines (resource issues – who pays to develop methods and maintain facilities?).

**Box 2:** Effects of antiangiogenic and antivascular therapeutics

**Antiangiogenic therapy**
• Inhibition of growth factor support of neovasculature.
• Reduced permeability.
• Reduced perfusion.
• Reduced blood volume.

**Antivascular therapy**
• Collapse of proliferating vasculature.
• Possible collapse of mature tumour vasculature.
• Loss of permeable vasculature.
• Reduced perfusion/flow.
• Reduced blood volume.
• Reduced tortuosity.

**Box 3:** Clinical issues

• Antiangiogenic therapies may have most effect on small tumours – not those traditionally monitored in phase I/II trials – challenging for MRI.
• In the absence of volume response, functional measurement information or biopsy may be required to gain the confidence to go to major phase III trials evaluating efficacy.
• What is the correct timing for measurements?
• To what extent can information from preclinical models guide clinical measurements?
• Can a dose-response be identified?

**Box 4:** Pharmaceutical industry view

• MR end points may aid selection of leads from many targets and therapeutic agents, for eventual phase III trials, reducing lead-time and costs.
• Ideally identifies markers of biological efficacy, guiding effective dose, scheduling and combinations.
• MR effect may not give a dose-response.
• Alternative methods of interest include PET, infrared, CT, U/S.
• Studies should be feasible, analysis should be robust.
• Approach should have widely accepted validity.
• Method should enable standardised implementation across several centres.
• A clear primary MR end point should be established prospectively.
• Type of analysis should be specified prospectively.
• There is a tension between industry preference for ICH GCP, academic preference for publication standard, the imperatives driving academic and clinical centres and the availability of funding to establish methodology to achieve these standards.

**Box 5:** Nomenclature and methods of analysis

**Nomenclature**
• Standardised terms should be employed as defined by Tofts *et al* (1999).
**Primary end points**
• *K*^trans^ or transfer constant reflects contrast delivery (perfusion) and transport across the vascular endothelium (permeability), with the dominant factor depending on whether delivery is flow or permeability limited. Values are derived by converting DCE-MRI data to contrast concentration, to which a pharmacokinetic model is fitted.
• IAUGC (initial area under the Gd concentration time curve) does not require a model, but does not have a simple relationship to physiology. It is a relatively robust and simple technique, although requiring quantitative T1 measurements.
**Secondary end points based on quantitative techniques**
• *v*_b_ (blood volume) can be derived from T2^*^ first-pass studies or from T1-weighted modelling techniques.
• *k*_ep_ is a function of *K*^trans^ and *v*_e_, describing contrast agent efflux from tumour.
• *v*_e_ (extravascular extracellular space (EES)) may reflect cellular density.
• Macromolecular contrast agents, although not yet recommended for trials of new therapeutic agents, are of value for preclinical experimental work. They can measure
• PS (endothelial permeability) and *v*_p_ (fractional plasma volume) without flow contamination.
• Measurements of PS may be hindered by low vascular leak into tumours, requiring long measurement times and showing poor signal to noise (particularly for T1-weighted measurements).

**Analysis**
• Model analysis should be based on the well-accepted Tofts or equivalent models, but with inclusion of arterial input normalisation, blood volume and classification of fit failures.
• Estimates of uncertainty should monitor model fitting and *χ*^2^ error, mapping this factor and including it in error analysis.
• Fit failures should be categorised as model fit failure (possibly multiple classes), no enhancement or noise.
**Data analysis**
• ROI or VOI analysis, based on whole tumour mean values, may not evaluate tumour heterogeneity, although it may be robust to motion. It may not reflect small areas of rapid change and so may be insensitive.
• Pixel mapping allows all data to be evaluated, allowing description and evaluation of regional change. Individual pixels will have relatively poor signal to noise.
• Analysis techniques, such as histogram and principal components analysis, may yield sensitive assessment of change.
• ROI placement needs to be supported by method of definition, and recorded to permit re-evaluation.

**Box 6:** Recommendations for MR measurement methods and end points for use in phase 1/2a trials of anticancer therapeutics

**Type of measurement**
• Pharmacodynamic assessment should use T1-weighted studies of low-molecular-weight Gd chelates.
• T2^*^-weighted studies may provide further information.
• Non-contrast-enhanced MRI may provide additional information.
• High-molecular-weight contrast agents may prove sensitive but are not yet recommended.
**Primary end points (terminology defined in Box 5)**
• The primary end point should be either *K*^trans^ (min^−1^)) or IAUGC (mMGd min).
• Vascularised tumour volume can be obtained by summing voxels with values above a predetermined threshold.
• Ideally, measurements of *K*^trans^ or IAUGC should be made for each voxel in the ROI or VOI.
• In tissues with substantial motion, ROI or VOI average measurements may be more appropriate.
• Three-dimensional measurements are preferred, as single-slice measurements (in theory) may be prone to bias due to incomplete sampling and errors in positioning the slice.
• Tumour volume should be measured.
• All data including ROI definition and analysis should be recorded and traceable to support external review.
**Measurement requirements to assess K^trans^ and IAUGC**
Both *K*^trans^ and IAUGC require calculation of instantaneous tumour Gd concentration, based on the change in relaxation rate due to contrast uptake ΔR1. This requires:
• An estimate of contrast agent relaxivity in tumour vasculature and tissue.
• Measurement of tumour T1 immediately prior to contrast uptake.
• An accurate T1 measurement method verified for all spatial locations, coils and scanners used.
• Cardiac output (or arterial input function).
• Reproducible injection (ideally power injector).
**Secondary end points**
• Simplified methods of characterising the contrast time curve in DCE-MRI are not recommended. They may be less sensitive than *K*^trans^ or IAUGC and are harder to compare between centres.
• Semiquantitative techniques are limited as they:
• May not accurately reflect tissue contrast agent concentration.
• May be influenced by contrast injection procedure, scanner settings, adjustment and coil behaviour.
• May be influenced by cardiac output.
• Have a poorly defined relation to physiology.
• Comparison between patients and between systems is difficult.
• Other end points derived from compartmental models and DCE-MRI such as *v*_b_, *v*_e_ and *k*_ep_ may be of value.
• More elaborate pharmacokinetic models may improve evaluation of dynamic data but are not yet supported by sufficient evidence to warrant use as primary end points.
• Non-DCE-MRI secondary end points include T1, T2, T2^*^, diffusion, perfusion from arterial spin tagging.
**Trial design**
• Entry criteria should consider tumour size in relation to pharmacological mechanisms, MRI resolution and potential confounding from rapid tumour growth rates.
• Investigators might consider dose escalation in individual patients, allowing each subject to act as their own control.

**Box 7:** Recommendations for analysis of DCE-MRI data in ROI or VOI

• Before placement of an ROI or VOI, individual images should be examined for the presence of patient motion, best seen on subtraction images.
• Ideally, dynamic image data sets should be spatially registered before analysis.
• Both early (60–120 s after contrast) and late (more than 5 min after contrast) subtraction images should be generated.
• Ideally, the early subtraction images will determine the position for ROI or VOI placement.
• If early enhancement is low, the late subtraction data set should be used.
• If no enhancement is seen, the baseline data (nonenhanced) aided by conventional images should be used for ROI or VOI placement.
• The outer limit of the lesion should act as a boundary of the ROI or VOI to minimise partial volume effects.
• Areas of necrosis and adjacent blood vessels should be excluded.
• The ROI or VOI should be constant in position and size for each image in the series under analysis.
• The position of the ROIs or VOIs, corresponding graphs and tables of enhancement values should be recorded, ideally in digital and hard copy form for future reference.
• In the event of significant motion, it may be necessary to adjust the ROI or VOI position on each image, measuring only a mean value.
• Analysis should take account of potential partial volume and ROI or VOI shape.

**Box 8:** Standardisation, validation and reproducibility

**Validation of MRI in relation to end points of action**
• Require correlation between size and type of biological effect and relevant MR parameter, in animal models, supported by clinical biopsy data.
• Time course of effects of rapidly acting agents needs to be defined.
• Require hypothesis-driven relationships between imaging and specific biological end points.
• Biological end points should relate to the mechanism of activity of the compound.
• It would be desirable to be able to predict the magnitude of the MR effect based on animal models, allowing trial design to monitor dose-related change.
**What validation/evidence of reproducibility is required ?**
• Centres should define reproducibility of data that is traceable, for individuals and intergroup comparisons, allowing the power of studies to be defined prospectively for a defined end point.
• Where possible, and in the absence of existing reproducibility data specific to the method, two baseline measurements should be incorporated to allow assessment of individual patient reproducibility.
• A standardised minimum statistical approach for reproducibility analysis should be defined.
**Standardisation of measurement methods**
• Basic standards for measurements of T1 should be established and adhered to. They should be tested against relevant phantoms, and reproducibility established.
• New techniques need to demonstrate specific advantages over existing methods, providing comparison data that define the benefit.
• In multicentre trials using identical (preferred), similar or different methods, comparison of precision and accuracy should be determined on phantoms, to provide a basis for pooling data, with account taken of correction for machine-specific factors, and for sensitivity to motion effects not seen in phantoms.
• Studies should include routine measurement and analysis quality assurance.
**Validation of analysis methods**
• Standardised data sets need to be made available to allow testing and comparison of analysis approaches.
• Research groups should make analysis methods available, either as open source code or by specific agreements where there are confidential or commercial issues.
• Standardisation of software for analysis would be desirable.
• Centres should be able to demonstrate that software is ‘fit for purpose’.
• Analysis of dynamic contrast-enhanced data in any multicentre trial should be performed at a single centre using validated software.
• Performance of measurements at each site should be validated at analysis site, prior to recruitment using standardised data from each site.

**Box 9:** Recommendations for future development

**Short term**
• Commercial equipment needs to provide rapid robust methods for measuring T1 as standard, with means of validation.
• Generally applicable methods of measuring arterial input function appropriate for all tumour sites of interest are required.
• Validated statistical tools for heterogeneity analysis are needed.
• A generally available database of anonymised standard DCE-MRI studies, with full information on the acquisition method and related diagnostic and clinical data, is desirable.
• More scientific work to define relationship between MR changes and action of therapeutics is required.
**Long term**
• Clinically applicable macromolecular contrast agents are required.
• Improved and more specific contrast agents for clinical use, including agents specifically designed for a given target/compound.
• Application of effective motion correction and registration techniques incorporated into measurement methods.
• Arterial spin tagging as an independent means of assessing perfusion should be investigated.
• The incorporation of simultaneous morphological, physiological and functional information into clinical studies may strengthen such investigations.
**Future requirements for clinical trials**
• Methods of supporting the MR developments required to underpin clinical trials need to be established.
• Trials using the MR techniques recommended here need the support of physicists and radiologists at all stages.
• For multicentre trials, this should include establishing and effecting cross-site standardisation of measurements and evaluation.

## Appendix

**Panel membership and author affiliations** Panel 1. End points and MR measurement methods Rapporteurs: IR Judson, JC Waterton Panel: JG Griffiths, MO Leach, GJ Rustin, GM Tozer, W Vennart, SR Williams

Panel 2. Analysis and nomenclature Rapporteurs: PS Tofts, AR Padhani Panel: JL Evelhoch, MV Knopp, D McIntyre, RJ Maxwell

Panel 3. Standardisation, validation and reproducibility Rapporteurs: A Jackson, W Vennart

Panel: AR Padhani, JC Waterton, MR Horsman, IR Judson, KM Brindle

Panel 4. Need for further development Rapporteurs: MO Leach, JR Griffiths Panel: JC Waterton, IR Judson, GC Jayson, P Price

Chairman of scientific session: P Workman

Organisers: MO Leach, R Rathbone

**Affiliations** KM Brindle, Department of Biochemistry, University of Cambridge, Tennis Court Road, Cambridge CB2 1GA, UK.

JL Evelhoch, Amgen Inc., One Amgen Center Drive, MS 38-4-C Thousand Oaks, CA 91320-1799, USA.

JR Griffiths, Department of Basic Medical Sciences, St George's Hospital Medical School, London SW17 ORE, UK.

MR Horsman, Department of Experimental Clinical Oncology, Aarhus University Hospital, Nörrebrogade 44, DK-8000 Aarhus, Denmark.

A Jackson, Imaging Science & Biomedical Engineering, G539 Stopford Building, University of Manchester Oxford Road, Manchester M13 9PT, UK.

GC Jayson, Cancer Research UK Department of Medical Oncology, Christie Hospital, Wilmslow Road, Withington, Manchester M20 4BX, UK.

IR Judson, Cancer Research UK Centre for Cancer Therapeutics, Institute of Cancer Research, 15 Cotswold Rd, Sutton, Surrey SM2 5NG, UK.

MV Knopp, Department of Radiology, Ohio State University Medical Center 657, Means Hall, 1654 Upham Drive, Columbus, OH 43210, USA.

MO Leach, Cancer Research UK Clinical Magnetic Resonance Research Group, Institute of Cancer Research, Royal Marsden Hospital, Downs Road, Sutton, Surrey SM2 5PT, UK.

RJ Maxwell, Gray Cancer Institute, Mount Vernon Hospital, PO Box 100, Northwood HA6 2JR, UK.

D McIntyre, Cancer Research UK Biomedical MR Group, St George's Hospital Medical School, Cranmer Terrace, Tooting, London SW17 0RE, UK.

AR Padhani, Paul Strickland Scanner Centre, Mount Vernon Hospital, Rickmansworth Road, Northwood, Middlesex HA6 2RN, UK.

P Price, Manchester Molecular Imaging Centre, University of Manchester, 27 Palatine Road, Manchester, M20 3LJ, UK.

R Rathbone, Drug Development Office, Cancer Research UK, London, UK.

GJ Rustin, Department of Medical Oncology, Mount Vernon Cancer Centre, Northwood, Middlesex, UK.

P Tofts, NMR Research Unit, Institute of Neurology, University College London, Queen Square, London, WC1N 3BG, UK.

GM Tozer, Tumour Microcirculation Group, Gray Cancer Institute, PO Box 100, Mount Vernon Hospital, Northwood, Middlesex HA6 2JR, UK.

W Vennart, Pfizer Central Research Department of ECRG (C746), Ramsgate Road, Sandwich, Kent CT13 9NJ, UK.

JC Waterton, Global Sciences & Information, AstraZeneca, Alderley Park, Macclesfield, Cheshire SK10 4TG, UK.

SR Williams, Imaging Science & Biomedical Engineering, Stopford Building, University of Manchester, Oxford Road, Manchester M13 9PT, UK.

P Workman, Cancer Research UK Centre for Cancer Therapeutics, Institute of Cancer Research, Haddow Laboratories, 15 Cotswold Rd, Sutton, Surrey SM2 5NG, UK.
